# Mucosal vaccine based on attenuated influenza virus and the group B Streptococcus recombinant peptides protected mice from influenza and *S*. *pneumoniae* infections

**DOI:** 10.1371/journal.pone.0218544

**Published:** 2019-06-25

**Authors:** Yulia Desheva, Galina Leontieva, Tatiana Kramskaya, Kornelia B. Grabovskaya, Vadim Karev, Andery Mamontov, Petr Nazarov, Alexander Suvorov

**Affiliations:** 1 Federal State Budgetary Scientific Institution "Institute of Experimental Medicine", Saint Petersburg, Russian Federation; 2 Saint Petersburg State University, Saint Petersburg, Russian Federation; 3 Federal State Budgetary Institution "Research institute of children's diseases", Saint Petersburg, Russian Federation; University of South Dakota, UNITED STATES

## Abstract

Although many influenza-related deaths are attributable to secondary bacterial infection with *S*. *pneumoniae*, vaccines that simultaneously protect against influenza and pneumococcal infection are currently not developed. The aim of our study was to evaluate the possibility to prevent post-influenza pneumococcal infection using an associated vaccine based on live influenza vaccine (LAIV) combined with recombinant polypeptides derived from superficial factors of Group B streptococcus (GBS) determining pathogenicity. We demonstrated in a model of post-influenza pneumococcal pneumonia that intranasal pneumococcal super-infection seriously complicated the course of A/Shanghai/2/2013(H7N9) CDC-RG virus infection in mice. Associated immunization using LAIV and GBS vaccine (GBSV) prevented post-influenza pneumococcal pneumonia better than mono-LAIV or GBSV immunization. At the same time, parenteral pneumococcal post-influenza infection of immune mice was more severe in the groups immunized using recombinant GBS peptides which can be explained by antibody-dependent enhancement of infection. In this case, the introduction of blockers of histamine receptors type 1 and 2 reduced the burden of secondary pneumococcal infection.

## Introduction

Many influenza-related deaths are attributable to secondary bacterial infection with *S*. *pneumoniae*, the most common cause of community-acquired pneumonia [1]. Since 2007, the pneumococcal serotypes not included in vaccines, begin to dominate and cause disease requiring the use of higher-valent vaccines [2, 3]. In addition, uncontrolled antibiotics consumption leads to the formation of resistant strains complicating the course of infection [4]. Polysaccharide components of pneumococcal vaccines do not provide long-term immunological memory, which requires their modification with adjuvants (Prevnar) or constant revaccination (Pneumo23).

In addition, capsular polysaccharide pneumococcal vaccine only partially protect against bacterial complications of influenza infection [5]. At the same time, influenza infection quite often leads to activation of conditionally pathogenic bacteria in the respiratory tract after influenza infection. *S*. *pneumoniae* invades its host by colonizing the naso-pharynx asymptomatically as it has been found to be part of the commensal microbiota of the upper respiratory tract [6, 7]. Influenza infection causes emergence of bacterial complications, mainly bronchitis and pneumonia, and leads to exacerbation of concomitant chronic diseases [8, 9]. The most well-known example is the common lethal synergy between influenza virus and pneumococcal or staphylococcal bacterial secondary infections [10].

Therefore, the aim of our study was to evaluate the possibility of preventing post-influenza pneumococcal infection by combining live influenza vaccine (LAIV) and recombinant polypeptides based on superficial factors of bacteria pathogenicity. In our previous research, it has been shown that the associated vaccine based on LAIV and recombinant group B streptococcus (GBS) polypeptides protects mice against post-influenza pneumonia following intranasal infection with GBS after homologous and heterologous influenza infection [11]. Here we evaluate potential of such an associated vaccine in preventing pneumococcal post-influenza infections in mice.

## Methods

### Viruses and vaccine preparations

The reassortant A/17/Mallard/Netherlands/00/95 (H7N3) was provided from Institute of Experimental Medicine collection of viruses. The A/Shanghai/2/2013(H7N9) CDC-RG virus was provided by Centers for the Diseases Control and prevention, USA. The viruses were propagated in CE and stored at -70°C. GBS recombinant polypeptides P6 (30-kDa), ScaAB (35-kDa) were expressed in *E*.*coli* and purified as described earlier [12].

### Pneumococci cultivation

*S*. *pneumoniae* clinical isolates № 73, serotype 3 or № 442, serotype 19F were used in this study were obtained from the collection of the Research Institute of Pediatric Infections (St. Petersburg, Russia). Pneumococci were cultured in anaerobic conditions at 37°C for 18 hours in THB medium with 20% horse serum (Becton Dickinson, New Jersey, USA). The Schaedler agar with sheep red blood cells was used as a solid medium for cultivation and counting of the bacterial number.

### Immunization of mice

The 8 to-10-week-old female DBA/2 mice were acquired from the laboratory breeding nursery of the Russian Academy of Sciences (Rappolovo, Leningrad Region, Russia). Groups of mice (60 animals in group) were lightly anesthetized with ether and intranasally (i.n.) vaccinated with 50 μL divided equally per nostril using the following preparations: 1) live influenza vaccine (LAIV) containing 1x10^7^ 50% egg infectious dose (EID_50_) of the A/H7N3 vaccine virus; 2) GBS vaccine (GBSV) containing the mix of P6 and ScaAB recombinant polypeptides (10 μg each, 20 μg total); 3) mixed LAIV+GBSV vaccine including 1x10^7^ EID50 of A/17/Mallard/Netherlands/00/95 (H7N3) virus and GBSV; 4) control animals were inoculated by PBS. The mice were immunized twice at an interval of 21 days. Three weeks after vaccination and revaccination, sera were collected from ether anesthetized mice via submandibular plexus. Nasal secrets were collected from mice after intraperitoneally administration of 0.1 mL of a 0.5% pilocarpine solution (Sigma-Aldrich, St. Louis, MO, USA) into the tubes containing 0.001 М of serine protease inhibitor phenylmethylsulfonyl fluoride (PMSF). Sera and nasal samples were stored at -20°C.

### Ethics statement

All procedures involving animals were performed according to the “Rules Laboratory Practice" Ministry of Health of the Russian Federation № 708 n. The study was approved by the Local Ethics Committee for Animal Care and Use at the Institute of Experimental Medicine, Saint-Petersburg, Russia. Non-terminal procedures were performed under ether anesthesia. Animals were euthanized by ether inhalation, and all efforts were made to minimize suffering of the animals. The body weight of the challenged mice was monitored and recorded once a day for 10 days post infection, and mice were euthanized if they lost more than 25% of starting body weight.

### Immunogenicity

Blood samples were taken from the submandibular vein. For hemagglutination-inhibition assay (HI) sera were treated with receptor-destroying enzyme (RDE, Denka Seiken, Tokyo, Japan) and tested for HI antibodies against A/17/Mallard/Netherlands/00/95 (H7N3) virus and against A/Shanghai/2/2013(H7N9) CDC-RG influenza as previously described [13]. The enzyme-linked immunosorbent assay (ELISA) was conducted to determine serum IgG and nasal IgA antibodies in 96-well microplаtes (Sarstedt AG & Co, Nümbrecht, Germany) as previously described [13]. For absorption we used 20 HAU/0.1 ml of the whole purified A/H7N3 virus or 20 HAU/0.1 ml of the whole purified A/Shanghai/2/2013(H7N9) CDC-RG virus or 0.2 mg/0.1 ml of GBSV individual components. The end-point ELISA titers were expressed as the highest dilution that yielded an optical density at 450 nm (OD_450_) greater than the mean OD_450_ plus 3 standard deviations (SD) of negative control wells.

### Interaction of immune sera with *S*. *pneumonia*

The dot -blot assay was performed using nitrocellulose membranes. Daily cultures of *S*. *pneumoniae* were washed in PBS and three microliters of each bacterial suspension were applied to nitrocellulose membranes and dried. The membrane was incubated in blocking buffer (5% dry milk dissolved in PBS pH 7.4). After the incubation, membrane was treated with mice sera diluted 1000 times in blocking buffer. Membrane was placed in a conjugate solution (anti-mouse IgG (Fc-specific)-peroxidase). Color was developed in ready to use TMB Liquid Substrate System for Membranes (Sigma-Aldrich, St. Louis, MO, USA) for 3–5 minutes.

For ELISA-test, the pneumococci of serotype 19F were absorbed on the surface of 96-well panels for serological reactions (Nalge Nunc International Corporation, Roskilde, Denmark) overnight. ELISA was performed as described above.

### Study of the protective efficacy of combined vaccination against secondary pneumococcal super-infection

On day 21 after revaccination the mice from all groups were inoculated intranasally with 300 fifty percent mouse infectious doses (MID_50_) of A/Shanghai/2/2013(H7N9) CDC-RG influenza virus. On 24 hours after viral infection the mice were intranasally inoculated with 50 ml of PBS containing 2.5x10^6^ CFU of *S*. *pneumonia* 3 serotype.

### Measurement of the viral titer and bacterial colonization in the lungs

To determine the viral titer in the lungs, the samples were collected from mice at 72 h after viral infection, homogenized in PBS containing 100 U/ml penicillin, 100 μg/ml streptomycin and centrifuged for 10 min at 6000 g. The viral titers were calculated as log10 of the fifty percent embryonic infectious doses (EID_50_) using hemagglutination as the endpoint, as described previously [14].

For measurement of bacterial numbers, lungs were removed from mice at 48 h after bacterial infection and homogenized in PBS using a Retsch MM-400 ball vibratory mill. Serial 10-fold dilutions of homogenates were made in PBS and aliquots of the dilutions were plated on dense nutrient medium (the Schaedler agar). Plates were incubated at 37°C in 5% CO2 for 14–16 hours before the colonies were counted under a microscope. The bacterial burden in colony forming units (CFU) per organ was calculated and expressed as a log 10.

### Histopathology

Lung sections obtained at 72 hours after primary viral infection or after 48 hours after pneumococcal invasion were fixed in 10% neutral buffered formalin and embedded in paraffin. Sections were stained with hematoxylin and eosin.

### Mouse model of systemic S. pneumoniae super- infection

On day 21 after revaccination the mice from all groups were inoculated intranasally with 300 MID_50_ of A/Shanghai/2/2013(H7N9) CDC-RG influenza virus and intraperinoneally injected with 325 CFU of *S*. *pneumoniae* 19F serotype 24 hours apart. Isolation of infectious streptococci from spleen was carried out on 48 hours after bacterial infection as stated above.

Simultaneously with secondary bacterial infection, half of the mice vaccinated using GBSV or LAIV+GBSV were injected intraperitoneally with a mixture of histamine receptor blockers H1 type (chloropyramine, “Egis”, Hungary) and H2 (famotidine, “Gedeon Richter”, Hungary), each at 6.7 mg / kg of weight, in a volume of 0.1 ml.

### Statistics

Data was processed using Statistica software, version 6.0 (StatSoft, Inc. Tulsa, Oklahoma, USA). Means and standard errors of means (SEM) were calculated to represent virus titers. Reciprocal antibody titers were expressed as log 2 (HI) or as log 10 (ELISA) and presented as mean±SEM. To compare two independent groups we used a Mann-Whitney U-test. To compare multiple independent groups we used a Kruskal-Wallis ANOVA test. The p-value <0.05 were considered to be statistically significant.

## Results

### Immunogenicity

**Fig 1:**
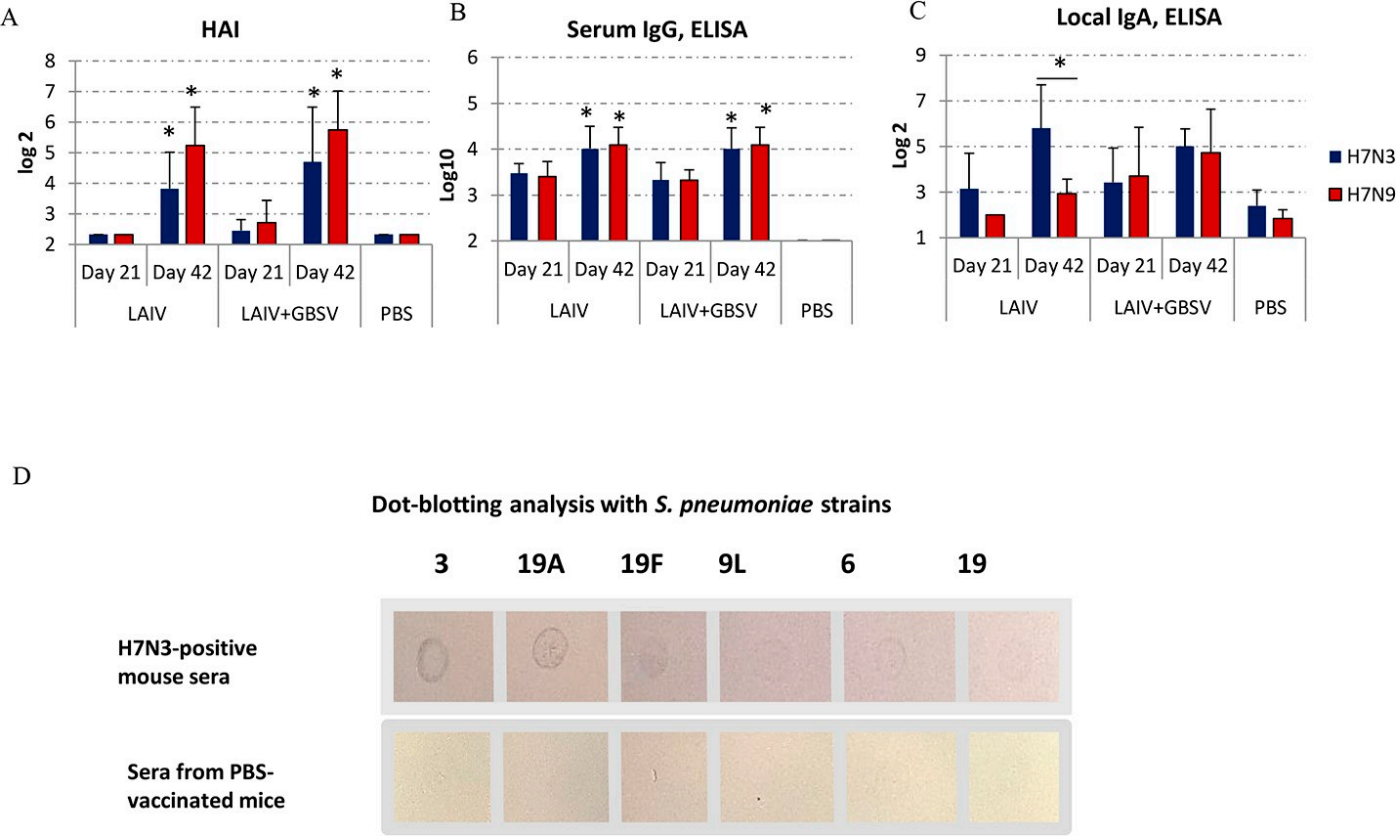
Immune response on day 21 and day 42 after intranasal immunization using LAIV or LAIV+GBSV (8 mice in each group, including PBS group). (A) serum HI antibodies against A/17/Mallard/Netherlands/00/95 (H7N3) and A/Shanghai/2/2013(H7N9) CDC-RG influenza viruses, *—P<0.005 compared to day 21. (B) serum virus-specific IgG, *—P<0.005 compared to day 21. (C) local virus-specific IgA, *—P<0.05. (D) dot-blotting analysis of pool sera obtained after LAIV-only immunization, reacting with *S*. *pneumoniae* strains.

Nasal immunization of DBA/2 mice using either A/H7N3 LAIV or combinations of the LAIV with two GBS polypeptides led to conversions of serum HI antibody conversions against the A/17/Mallard/Netherlands/00/95 (H7N3) strain (Fig 1A).

The introduction of two-doses of LAIV or associated LAIV+GBSV vaccine was required to achieve HI antibody response. The titers of HI antibody against heterologous A/Shanghai/2/2013(H7N9) CDC-RG virus were even higher than that of the homologous virus. At the same time, local IgA response was more pronounced against homologous A/17/Mallard/Netherlands/00/95 (H7N3) than against A/Shanghai/2/2013(H7N9) CDC-RG (Fig 1C). We detected serum IgG in equal titers against either whole A/17/Mallard/Netherlands/00/95 (H7N3) or A/Shanghai/2/2013(H7N9) CDC-RG influenza viruses (Fig 1B). Unexpectedly, the sera of LAIV-immunized mice reacted with different serotypes of pneumococci. As seen in Fig 1D sera, positive for the A/17/Mallard/Netherlands/00/95 (H7N3) virus cross-reacted with various pneumococcal serotypes according to the results of dot-blot analysis. In the sera of control mice, no such response was detected (Fig 1D). Since this experiment we used a pool of sera, the significance of differences was not established.

**Fig 2:**
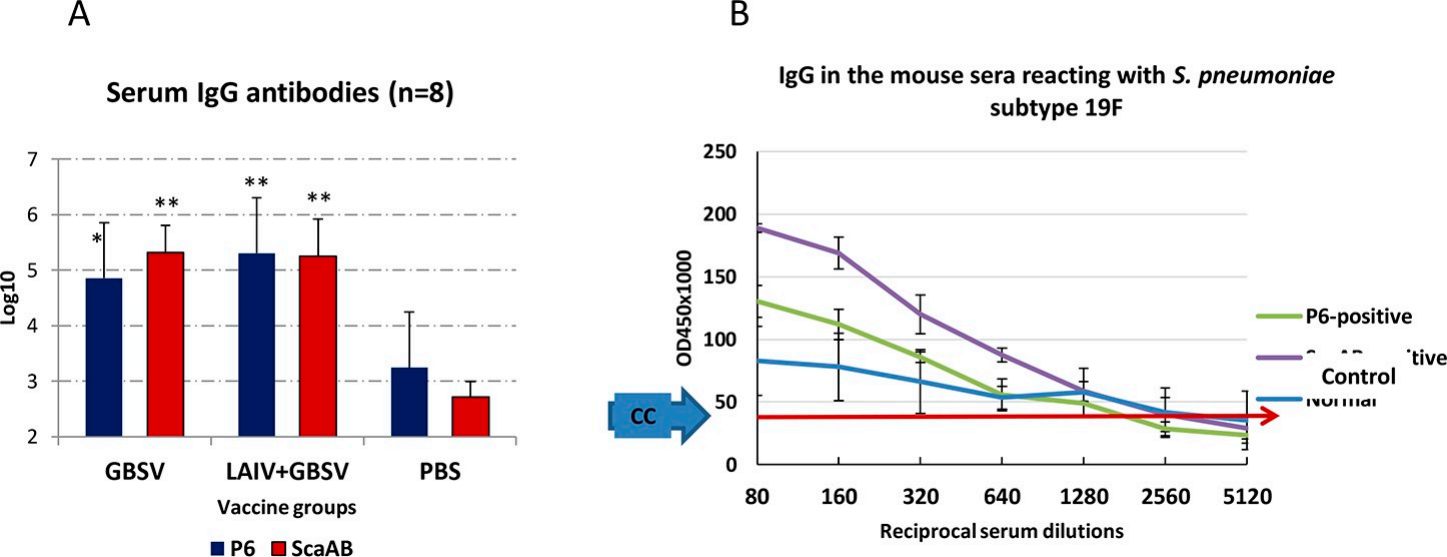
Immune response against GBS components or *S*. *pneumoniae* 19F serotype after intranasal immunization using GBSV or LAIV+GBSV. (A) serum IgG specific to GBS recombinant peptides P6 and ScaAB on day 42 after first vaccination (ELISA); *—P < 0.01, **—P < 0.001 compared to PBS group. (B) plot of serial serum dilution reacting with precipitated *S*. *pneumoniae* serotype 19F (ELISA); each curve represents data from six tested sera; the arrow represents the control level obtained as mean OD_450_ of 5–8 negative wells plus 3 SD.

Serum IgG, specific to GBS polypeptides P6 and ScaAB was detected on day 21 and 42 post primary immunization in titers significantly higher compared to controls (Fig 2A).

Intranasal immunization using GBSV or LAIV+GBSV raised IgG not only against GBS components P6 and ScaAB (Fig 2A), but also against *S*. *pneumoniae* immobilized on the wells surface of plates for immunological reactions (Fig 2B). In this case, the reaction of ScaAB-positive sera was more pronounced compared to reaction of P6-positive with *S*. *pneumoniae* of serotype 19F (Fig 2B).

### Isolation of infectious virus and streptococci from the lungs and weight loss after viral-bacterial challenge

**Fig 3:**
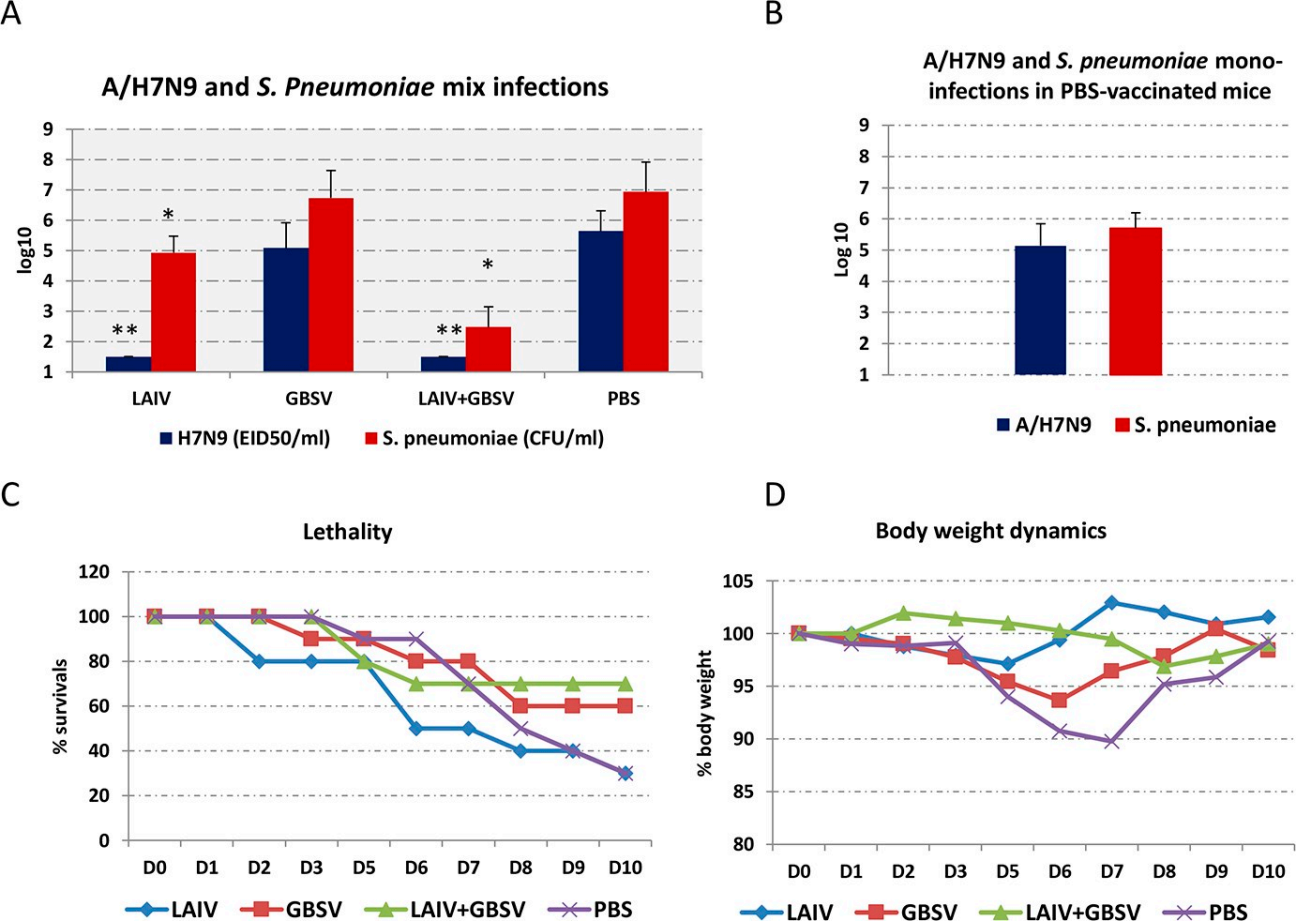
The A/Shanghai/2/2013(H7N9) CDC-RG influenza virus and *S*. *pneumoniae* serotype 3 isolation from the lungs. (A) Influenza virus and *S*. *pneumoniae* titers in the lungs with mixed virus-bacterial infection at 72 hours after primary viral infection (n = 6–8); *—P < 0.01, **—P < 0.001 compared to mock-vaccinated animals. (B) Influenza virus and *S*. *pneumoniae* titers in the lungs of mock-vaccinated animals at 72 hours after A/H7N9 mono-infection or after 48 hours after *S*. *pneumoniae* serotype 3 mono-infection (n = 6). (С) lethality after mixed influenza and pneumococcal infections (n = 10)/ (D)–body weight dynamics after mixed influenza and pneumococcal infections (n = 10).

After intranasal post-influenza pneumococcal infection, the infectious virus A/Shanghai/2/2013 (H7N9) CDC-RG was not isolated from the lungs of mice immunized using LAIV, and this may result in a 100-fold reduction of secondary bacterial infections (Fig 3A).

The associated virus-bacterial vaccine prevented not only viral infection of the lungs, but also reduced pneumococcal titers by 25,000x (Fig 3A). Thus, mixed vaccination was most effective in reducing bacterial lung titers during secondary bacterial infection.

Combined virus-bacterial infection led to increased lung titers of both infectious virus and infectious pneumococcus in the lungs. As can be seen in Fig 3B, both viral and bacterial load in mock-vaccinated mice after mixed infections was higher compared with mono-infections, although these differences were not statistically significant.

Although mono- infection with A/Shanghai/2/2013(H7N9) CDC-RG influenza virus was not lethal for mice, 70% of mock-vaccinated animals died after super-infection with a non-lethal dose of bacteria (Fig 3C). Under conditions of super-infection, LAIV did not protect against mortality compared to super-infected PBS controls. However, unlike intact mice, the surviving mice in this group did not lose weight (Fig 3D). These data, together with decreased bacterial lung titers, allow us to conclude that LAIV created a partial defense against post-influenza pneumococcal infection. Vaccines containing recombinant peptides of GBS provided the best protection from mortality after pneumococcal super-infection with 70% of the mice surviving in the case of LAIV+GBSV immunization, and 60% surviving in GBSV vaccine group (Fig 3C).

### Pathomorhological data

**Fig 4:**
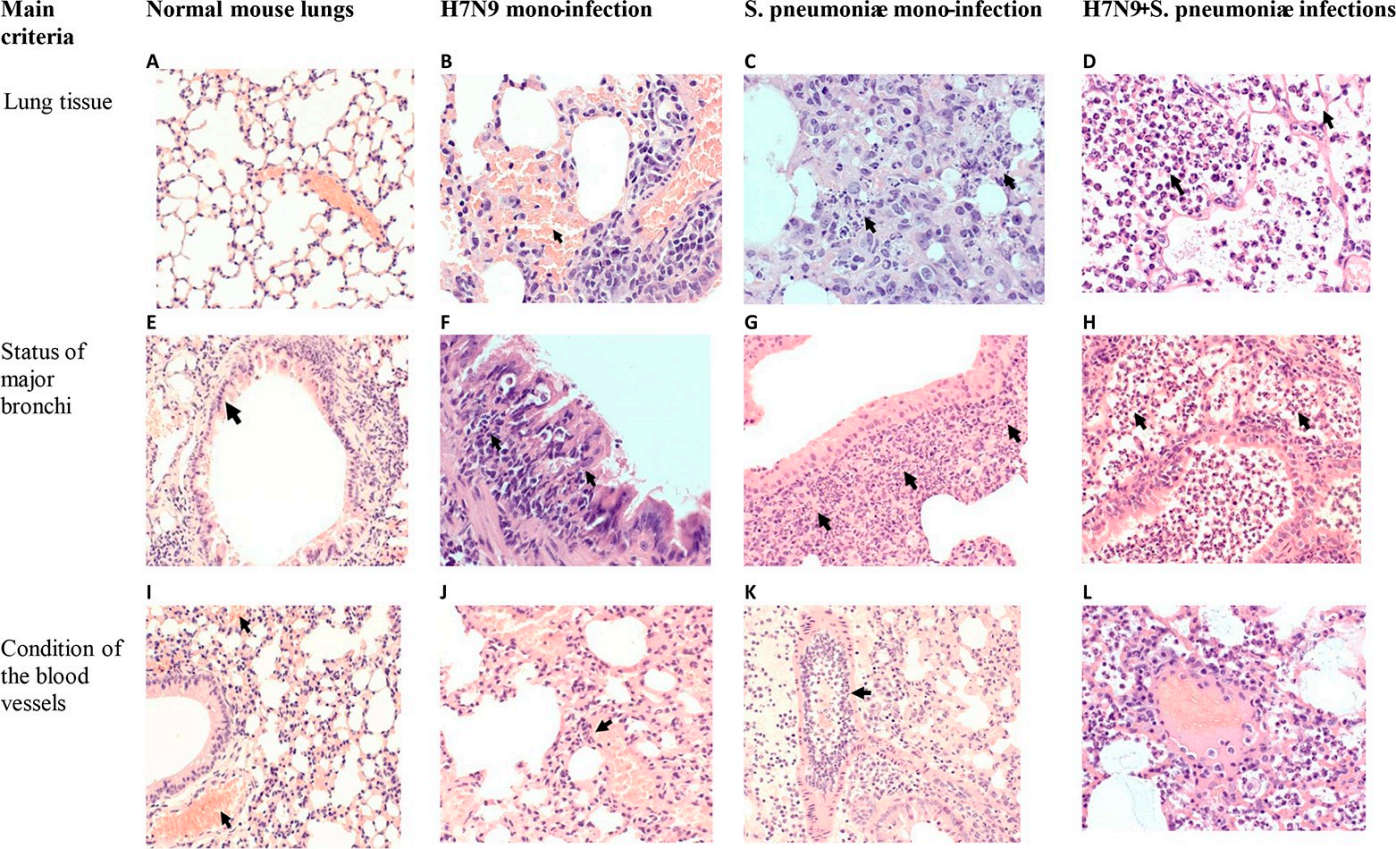
Hematoxylin-eosin stained sections from lung tissues of mice at 3 days after A/Shanghai/2/2013(H7N9) CDC-RG infection and at 48 hours after *S*. *pneumoniae* infection or at 48 hours after serial viral-bacterial infections. (A,B,C,D,F,K,L) Magnification, ×400. (E,G,H,I,J) Magnification, ×200. The meanings of the arrows are indicated in the text.

Data on the isolation of infectious virus and pneumococci from the lungs were confirmed by histological examination of hematoxylin-eosin stained lung sections. The evaluation of pathomorphological signs of post-influenza pneumococcal infection involved a comparison with a mono-infection with the A/Shanghai/2/2013(H7N9) CDC-RG influenza virus or pneumococcal infection. In non-treated mice, lung tissue structure was normal (Fig 4A) with the exception of acute non-specific microcirculation disorders.

These changes comprised clusters of a small amount of red blood cells in the lumen of part of the alveoli in the sub-pleural regions and acute plethora (Fig 4I). The epithelial lining of the major bronchi was preserved, the epithelium was single-row, of normal structure, and there were no contents in the lumen or cell infiltration of the bronchial walls (Fig 4E). Acute exudative bronchitis developed at 72 hours after viral mono-infection, with accumulations of a small amount red blood cells in part of alveoli lumen (Fig 4B, arrowhead), acute plethora and marginal leukocyte was standing in the lumen of blood vessels (Fig 4J, arrowhead). In the lumen of the large bronchi leukocyte exudate (Fig 4F), the integrity of the mucous membrane was preserved over a large extent, in some areas with focal leukocyte infiltration of the epithelial layer (arrowhead).

Focal bacterial bronchopneumonia developed after pneumococcal mono-infection (Fig 4C, 4G and 4K). Disintegrating polymorphonuclear leukocytes were detected in pneumonic foci, when lymphocytes were observed among desquamated cells of the alveolar epithelium (Fig 4C, arrowhead). In the lumen of a part of the large bronchi single polymorphonuclear leukocytes were detected, the epithelium was damaged, with the formation of multi-row, peribronchial polymorphoncellular infiltration followed by a significant admixture of polymorphonuclear leukocytes (Fig 4G, arrowhead).

When pneumococci were administered intranasally at 24 hours after the viral infection, the signs of macrofocal bacterial pleuropneumonia were registered on day 2 after bacterial infection (Fig 4D, 4H and 4L). Therefore, co-infection greatly increased the severity of the morphological changes in the lungs.

**Fig 5:**
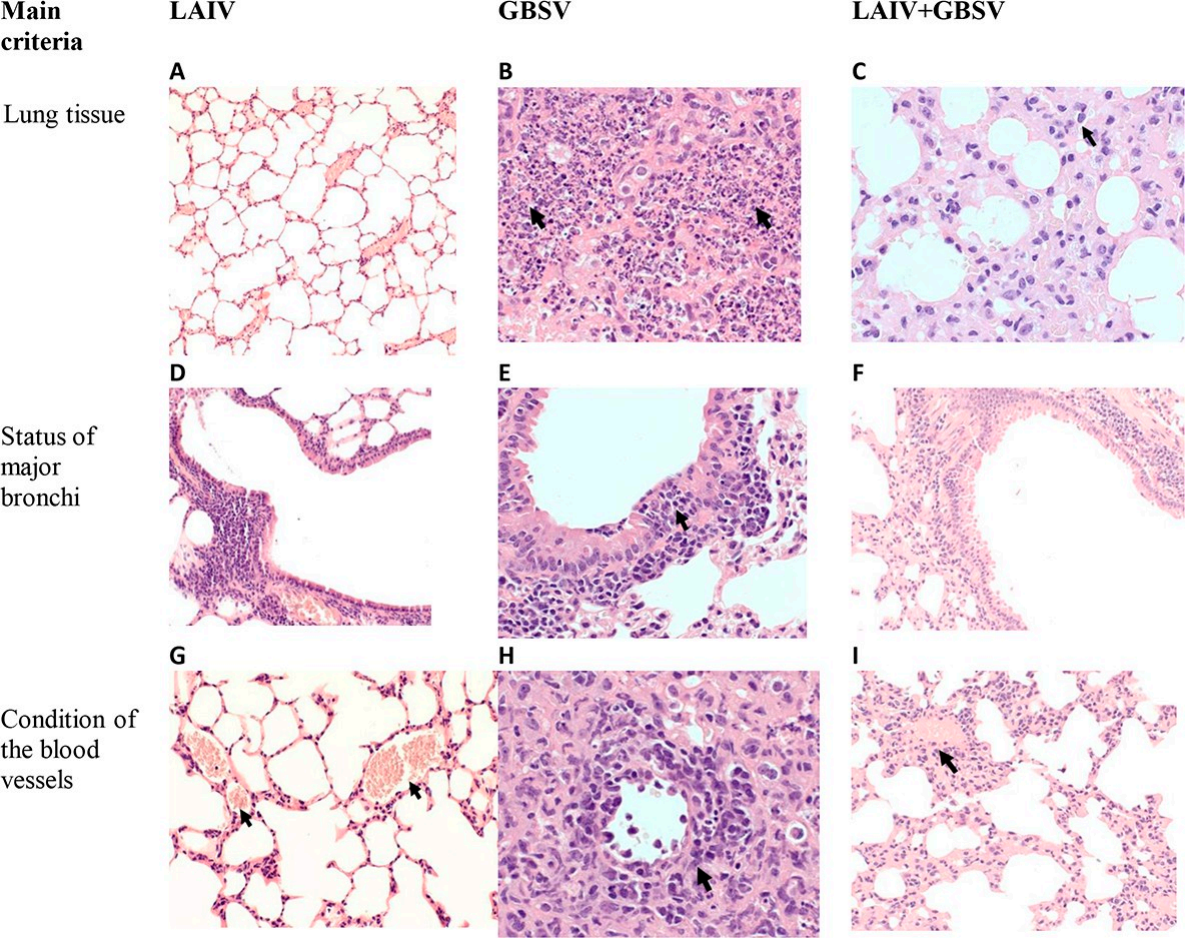
Comparative study of pathological changes in the lungs of mice at 48 hours after serial A/Shanghai/2/2013(H7N9) CDC-RG *and S*. *pneumoniae* infections. (A) Magnification, ×100; B,C,E,Y– 400. (D,F,G,I)Magnification, ×200. The meanings of the arrows are indicated in the text.

Sequential viral-pneumococcal intranasal infection in LAIV-immunized mice caused focal serous-desquamative pneumonia (Fig 5A) with small areas of serous pneumonia, desquamated cells of the alveolar epithelium, single polymorphonuclear leukocytes, lymphocytes and erythrocytes found in the lung tissue.

There were no contents in the lumen of the large bronchi; the bronchus wall was of normal structure (Fig 5D).

After GBSV immunization, sequential viral-bacterial challenge lead to large focal, partly confluent destructive pleuropneumonia, when the formation of small foci of purulent destruction was observed (Fig 5B, arrowhead) along with focal leukocyte infiltration of the medium bronchi mucous membrane (Fig 5E, arrowhead) and severe damage to the vascular endothelium (Fig 5H, arrowhead). That is, the changes were characteristic of a virus-bacterial infection.

After associated LAIV+GBSV vaccination, focal serous and hemorrhagic pneumonia prevailed in conditions of post-influenza bacterial super-infection (Fig 5C, arrowhead). In this case, the walls of the major bronchi retained their usual structure (Fig 5F), although widespread perivascular polymorphocellular infiltration and pronounced damage to the vascular endothelium were noted (Fig 5I, arrowhead). Thus, bacterial super-infection complicated the morphological pattern of lung lesions compared with viral or bacterial mono-infection, despite the fact that there was no significant increase in viral and bacterial reproduction. After immunization with LAIV or mixed viral-bacterial immunization, lung damage was limited to hemorrhagic and serous changes, while immunization with GBS did not prevent viral-bacterial damage to the lungs, as was established by intranasal post-influenza pneumococcal super-infection. We also attempted to study how the associated virus-bacterial immunization will act against the generalized post-influenza pneumococcal infection.

### Systemic post-influenza pneumococcal infection of immunized mice

**Fig 6:**
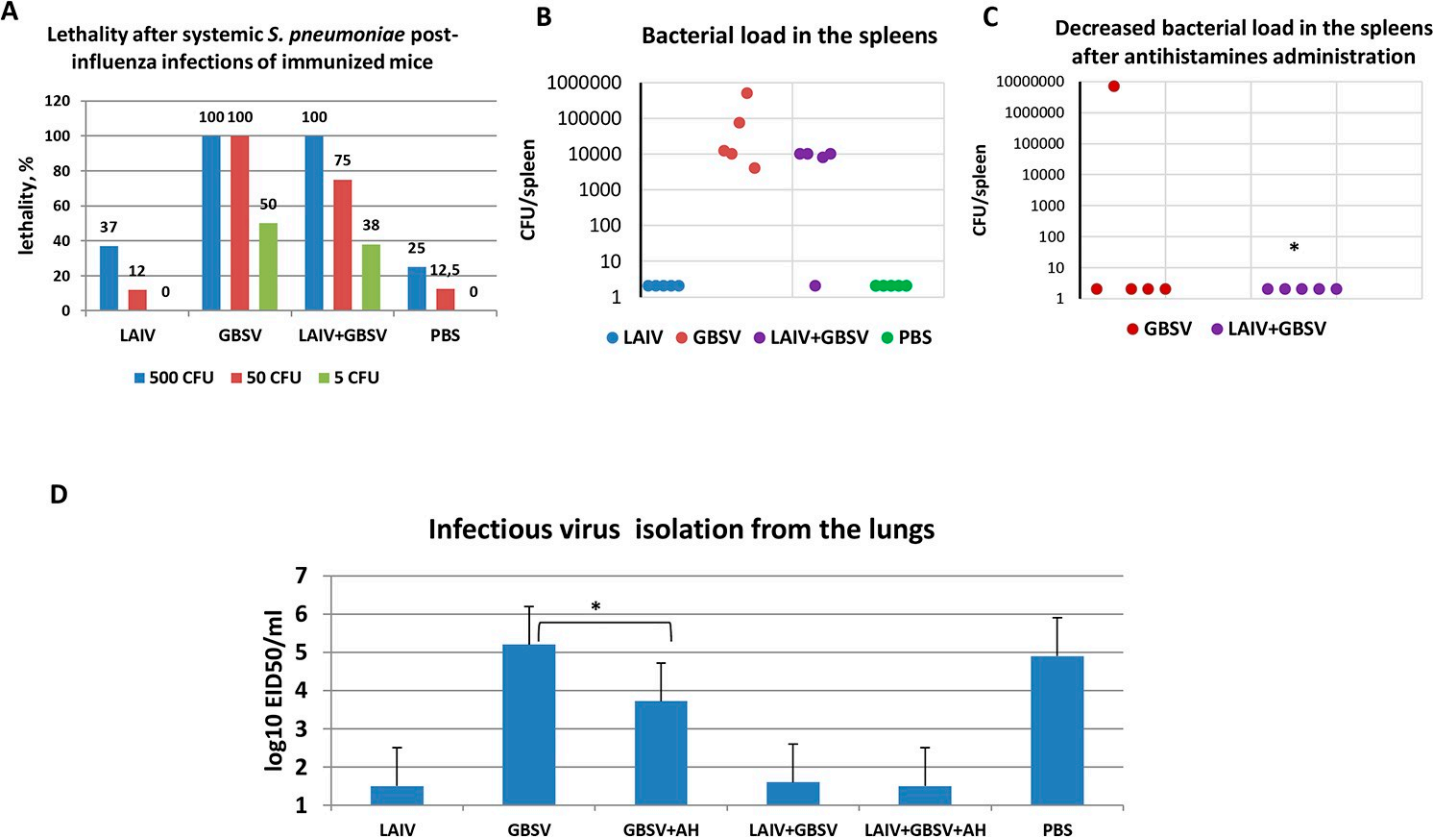
Intranasal challenge of immunized mice with A/Shanghai/2/2013(H7N9) CDC-RG followed by parenteral super-infection 24 hours apart with *S*. *pneumoniae* serotype 19F. (A) Lethality at 24 hours after parenteral post-influenza infection with different doses of pneumococci in groups of immunized mice (n = 8). (B) Bacterial load in the spleens 48 hours after post-influenza parenteral pneumococcal infection with 325 CFU (n = 5). (C) Decreased bacterial load in the spleens after antihistamines (AH) administration (n = 5). *- P = 0.036 compared to pneumococcal titers with no AH administration. (D) Infectious virus isolation from the lungs (“AH” means antihistamine administration) 24 hours after post-influenza parenteral pneumococcal infection (n = 5); *—P = 0.009.

*S*. *pneumoniae* were administered intraperitoneally to reproduce systemic post-influenza pneumococcal infection in mice. Unlike intranasal pneumococcal superinfection, after systemic *S*. *pneumoniae* super-infection we observed increased mortality at 48 hours among mice that received GBSV or LAIV+GBSV (Fig 6A).

In GBSV or LAIV+GBSV groups, there was an increased isolation of pneumococci from the spleens (Fig 6B). The introduction of histamine blockers significantly reduced the infecting pneumococci isolation from the spleen of mice (Fig 6B). Parenteral administration of antihistamines also reduced the release of infectious virus from the lungs in the group of mice immunized with streptococcal peptides (Fig 6D). These data allow us to conclude that a decrease in infection of internal organs after introduction of chloropyramine and famotidine can be caused by the abolition of the impact of histamine on the vascular hiperpermeability and inhibition of the development of sepsis.

## Discussion

Live attenuated influenza vaccines are of particular importance for the prevention of pandemic and potentially pandemic influenza due to formation of systemic and strong local (secretory) immunity. Intranasal vaccine implementation produces immune response similar to natural infection, and therefore induces an early, broad and long–lasting protection [15]. The cost of live vaccine is several times lower compared to inactivated vaccines, and the capacity of the LAIV biotechnological production process is significantly higher [15].

Peptide vaccines based on superficial factors of bacteria (highly conservative lipoprotein virulence factors, enzymes such as nucleases, proteases, hemolysin, peptidase) are preferable over polysaccharide vaccines due to the smaller degree of variability and higher immunogenicity. That is why we decided to study immunogenicity and protection against pneumococcal infection of ScaAB, which is the major surface lipoprotein of GBS closely related to PsaA protein of *S*. *pneumoniae* [16]. PsaA protein is considered to be the most promising vaccine candidate against *S*. *pneumoniae* infection [17]. Recently, Seon et al., 2017 have shown that attenuated pneumococcus pep27 mutant which protect mice against secondary pneumococcal challenge after influenza virus infection increased antibody titers against PspA protein but not type 2 capsular polysaccharide These data suggest that although pneumococcus is surrounded by a powerful polysaccharide capsule, at the stages of its reproduction, the internal proteins become available for molecular interaction and the antibodies forming against these proteins are protective [18].

When mice were immunized with LAIV or LAIV in combination with GBSV, the immune response of anti-hemagglutinating antibodies was observed not only to the homologous A/H7N3 virus, but also to the heterologous A/H7N9. Data of HI analysis confirm previously obtained data on outbred mice about a higher immune response of HI antibodies against A/H7N9 viruses after immunization with A/H7N3 vaccine [19].

We showed that the sera of mice, positive for the influenza virus, reacted with pneumococci of various serotypes, including 3 and 19F, which were later used for intranasal and parenteral infection, respectively. This phenomenon presumably can be explained by receptor interactions, as previously it has been demonstrated *in vitro* that HA of influenza viruses A/H1N1 and A/H3N2 recognizes the α2,6-linked sialic acid present in the capsular polysaccharide of *Streptococcus suis* [20].

Data on the formation of serum IgG against streptococcal polypeptides confirm the evidence that polypeptides cause a pronounced systemic immune response when administered intranasally. The data obtained on the model of systemic pneumococcal post-influenza infection serve as an additional confirmation of the high immunogenicity of streptococcal peptides after intranasal administration. In this case, the mortality of mice was higher in those groups that were immunized using streptococcal peptides and had serum IgG against ScaAB, which is similar to PsaA protein of *S*. *pneumoniae*. This was confirmed in ELISA-test when ScaAB-positive sera reacted with *S*. *pneumonia* 19F serotype rather than P6-positive sera (Fig 2B).

Previously, it was shown that live attenuated influenza vaccines may significantly reduce bacterial disease during influenza epidemics [21]. In our study, the introduction of LAIV had a positive effect on the course of a virus-bacterial infection, when viral replication and bacterial infection in the lungs after challenge was lower compared to non-vaccinated animals (Fig 3B). It is noteworthy, that the decrease in bacterial lung infection after LAIV vaccination was even more pronounced than after the administration of GBSV alone. Of all vaccine preparations, only the associated vaccination using LAIV+GBSV resulted in the most significant reduction in lung bacterial infection. It can be assumed, that this effect may be associated not only with the formation of antibodies, but also with a decrease in primary influenza infection.

The results of quantitative viral and bacteriological studies corresponded to the morphological features in the lungs which suggest that immunization with LAIV+GBSV was the most successful in reducing the severity of pneumococcal post-influenza super-infection. It is noteworthy that GBSV alone did not prevent the virus-bacterial pulmonary lesions and body weight decrease, although at the same time GBSV protected against mortality an additional 30% of mice. This presumably can be explained by the presence of the ScaAB peptide in the preparation.

The mechanisms of partial protective effect against streptococcal infection after LAIV vaccination may be associated with the processes of bacterial adherence. One of possible reasons for changing the sensitivity of tissues to bacterial load associated with viral reproduction is that the virus can change peptide profile of the cell membrane and form unusual for intact cell receptor sites. The receptor sites for bacteria may be formed on the cell membrane by displacing host proteins by viral polypeptides. So, it was previously shown that the transfer of HA-specific CD8 T cell clones in transgenic mice with expressed HA antigen on the alveolar epithelial cells leads to progressive lethal lung injury in the absence of active viral reproduction [22]. The second most important antigenic component of the influenza virus, neuraminidase, as a part of an influenza virus may nonspecifically bare receptor located in the membrane of epithelial cells [23].

When simulating intranasal bacterial super-infection, the associated virus-bacterial immunization protected better than mono-LAIV or GBSV. However, when infected with pneumococci, the development of systemic spread cannot be ruled out [24], especially against the background of primary influenza infection. In this regard, we reproduced secondary systemic pneumococcal infection in mice immunized with various preparations through peritoneal infection. It was unexpectedly found that increased levels of IgG antibodies to GBS peptides in groups of mice immunized with GBSV were associated with the development of severe systemic infection. The problems of antibody-dependent enhancement (ADE) are widely discussed in scientific literature, including discussions on vaccine development issues. ADE syndrome is characteristic of dengue fever, in which it initially attracted attention and was first described [25], but also is observed in other infections [26, 27]. One of the factors contributing to ADE development is considered to be the "noncanonical" mechanism of antigen presentation, due to the involvement in the presentation process of many cells expressing Fcγ receptors (CD16, CD32, CD64) and activated by IgG-containing immune complexes, in particular B-cells [28].

In conclusion, it should be said that using a mouse model, we demonstrated that bacterial pneumococcal infection seriously complicates the course of A/H7N9 influenza infection. Evidence has been obtained that associated immunization with the use of LAIV and recombinant peptides of GBS is capable to prevent post-influenza pneumonia. Data on the positive effect of LAIV on the course of post-influenza pneumococcal pneumonia are especially important in light of the fact that LAIV is widely used in a number of countries for the vaccination of children [29]. At the same time, generalized infection of immune mice was more severe in the groups immunized using recombinant GBS peptides which can be explained by antibody-dependent enhancement. In this case, the introduction of histamine receptors blockers of type 1 and 2 reduced the burden of secondary pneumococcal infection -.

In mice with high titers of IgG antibodies against GBS peptides, we observed a high pneumococcal colonization of organs and an increase in lethality on 48 hours, i.e. signs of bacteremia and septic shock. We assume this can be attributed to activation of mast cell by IgG-containing immune complexes. After parenteral infection, the bacteria immediately met with a large excess of circulating antibodies. In this case, the must cells activation should followed by a massive release of histamine. This caused a systemic vascular hyperpermeability with the vascular leak and expansion of bacterial colonization of organs. These processes could be aggravated by developing influenza infection.

Antihistamines in our experiments interrupted the development of sepsis by blocking the vascular hyperpermeability after histamine release [30]. These data indicate that during the reinfection of immune persons, the immunocomplex pathway of mast cell activation by IgG-containing immune complexes should be taken into account and carefully studied. In particular, the dependence of the phenomenon of increasing bacterial contamination of internal organs and increasing mortality on the dynamics of IgG antibodies (the timing of their appearance, the rate of increase in concentration, isotypic composition) should be studied in experimental models. Anyway, the ADE-phenomenon should be studied when immunizing not only against dengue or Zika viruses [31, 32], but also in influenza and pneumococcal immunization. We consider our data as a contribution to this topic.

## Supporting information

S1 Appendix S. Pneumoniae in the lungs of mice after bacterial super-infection. (PDF)

## Abbreviations

ADE: Antibody-Dependent Enhancement
EID_50_: Fifty percent Egg Infectious Doses
ELISA: Enzyme-linked immunosorbent assay
GBS: Group B *Streptococci*
GBSV: Group B *Streptococci* protein vaccine
GMTs: Geometric Mean Titers
HI: Hemagglutination-inhibition assay
LAIV: Live influenza vaccine
MID_50_: Fifty percent Mouse Infectious Doses
PMSF: Phenylmethylsulfonyl Fluoride
SEM: Standard errors of the means
