# Supplementary figures and images for: Mucosal vaccine based on attenuated influenza virus and the group B Streptococcus recombinant peptides protected mice from influenza and S. pneumoniae infections
Source: PLoS One. 2019 Jun 25;14(6):e0218544. doi: 10.1371/journal.pone.0218544 (PMC6592537; doi:10.1371/journal.pone.0218544)

## S. Pneumoniae in the lungs of mice after bacterial super-infection.

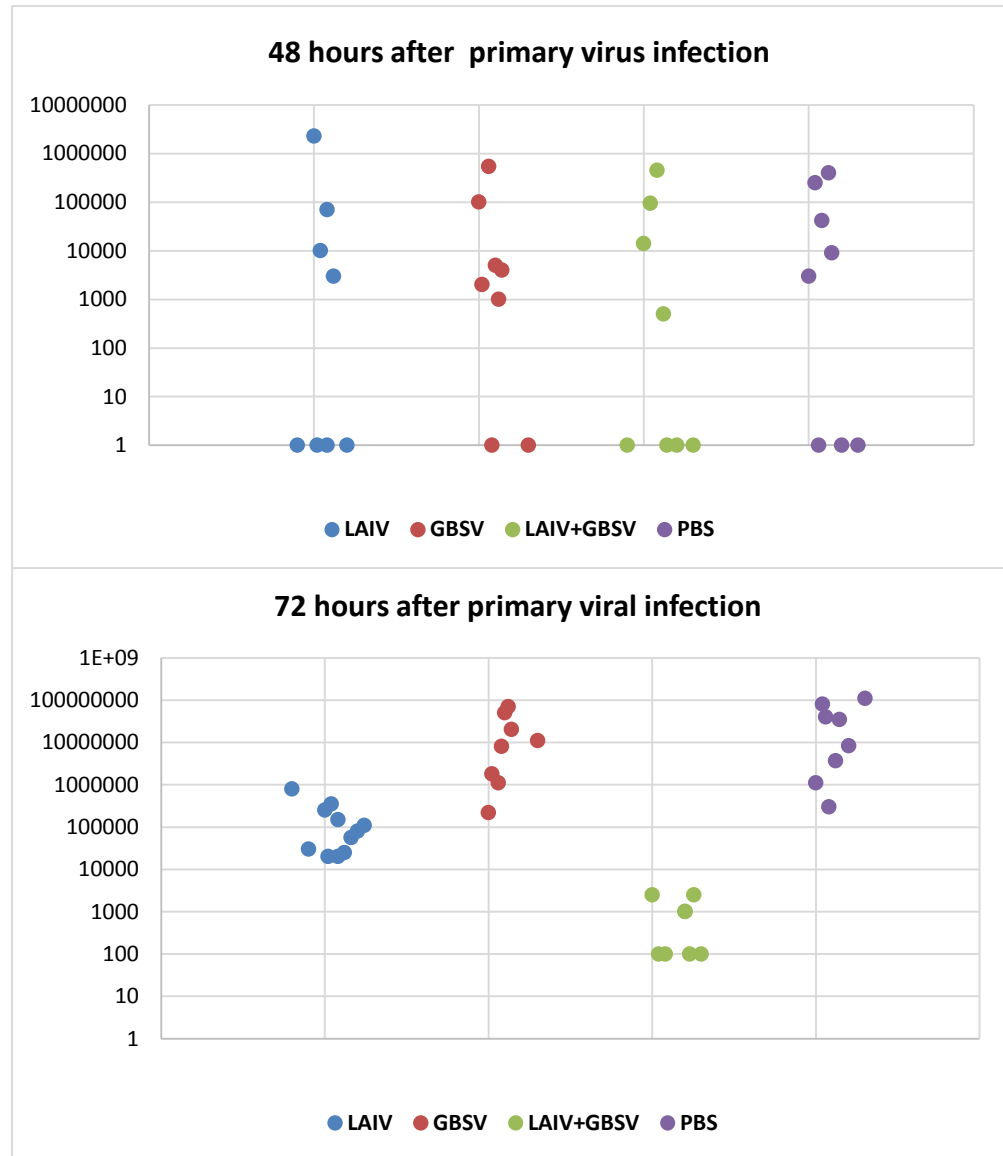

Supplement: S1 Appendix — (PDF) [file pone.0218544.s001.pdf]
